# Screening persistent organic pollutants for effects on testosterone and estrogen synthesis at human-relevant concentrations using H295R cells in 96-well plates

**DOI:** 10.1007/s10565-024-09902-4

**Published:** 2024-08-13

**Authors:** Denise Strand, Erik Nylander, Andrey Höglund, Bo Lundgren, Jonathan W. Martin, Oskar Karlsson

**Affiliations:** 1 Science for Life Laboratory, Department of Environmental Science, Stockholm University, 114 18 Stockholm, Sweden; 2 Science for Life Laboratory, Biochemical and Cellular Assay unit, Dept. of Biochemistry and Biophysics, Stockholm University, 106 91 Stockholm, Sweden

**Keywords:** Steroidogenesis, Endocrine disruption, POPs, H295R, OECD TG#456, Exposome

## Abstract

Many persistent organic pollutants (POPs) are suspected endocrine disruptors and it is important to investigate their effects at low concentrations relevant to human exposure. Here, the OECD test guideline #456 steroidogenesis assay was downscaled to a 96-well microplate format to screen 24 POPs for their effects on viability, and testosterone and estradiol synthesis using the human adrenocortical cell line H295R. The compounds (six polyfluoroalkyl substances, five organochlorine pesticides, ten polychlorinated biphenyls and three polybrominated diphenyl ethers) were tested at human-relevant levels (1 nM to 10 µM). Increased estradiol synthesis, above the OECD guideline threshold of 1.5-fold solvent control, was shown after exposure to 10 µM PCB-156 (153%) and PCB-180 (196%). Interestingly, the base hormone synthesis varied depending on the cell batch. An alternative data analysis using a linear mixed-effects model that include multiple independent experiments and considers batch-dependent variation was therefore applied. This approach revealed small but statistically significant effects on estradiol or testosterone synthesis for 17 compounds. Increased testosterone levels were demonstrated even at 1 nM for PCB-74 (18%), PCB-99 (29%), PCB-118 (16%), PCB-138 (19%), PCB-180 (22%), and PBDE-153 (21%). The MTT assay revealed significant effects on cell viability after exposure to 1 nM of perfluoroundecanoic acid (12%), 3 nM PBDE-153 (9%), and 10 µM of PCB-156 (6%). This shows that some POPs can interfere with endocrine signaling at concentrations found in human blood, highlighting the need for further investigation into the toxicological mechanisms of POPs and their mixtures at low concentrations relevant to human exposure.

**Graphical Abstract:**

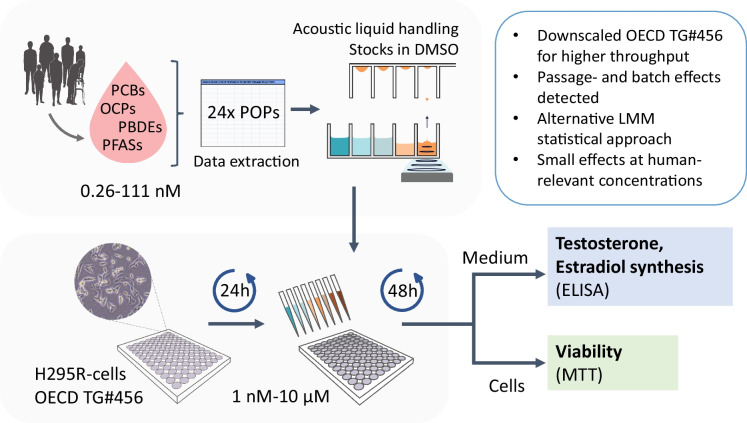

**Supplementary Information:**

The online version contains supplementary material available at 10.1007/s10565-024-09902-4.

## Introduction

Many persistent chemical contaminants are omnipresent in the environment, leading to chronic human exposure via inhalation, ingestion, and dermal contact from food, consumer products, air or dust (Encarnação et al. 2019; Karlsson 2023; La Merrill et al. 2013). Some are classified as persistent organic pollutants (POPs) due to their long environmental half-lives, bioaccumulation, and potential toxicity (Encarnação et al. 2019). POPs include polyfluoroalkyl substances (PFAS), organochlorine pesticides (OCPs), polychlorinated biphenyls (PCBs) and polybrominated diphenyl ethers (PBDEs). They have all been detected in numerous human tissues and body fluids (Björvang et al. 2021; Encarnação et al. 2019).

There is a growing concern regarding the harmful potential of POPs in humans. At the low levels detected in human tissue, there is little risk of acute toxicity. However, sensitive and subtle adverse effects that may only be visible over time at a population level due to chronic exposure are difficult to detect and prevent (Encarnação et al. 2019; van Duursen et al. 2020). This includes endocrine disruption, which can cause adverse effects on human and organismal development, brain function, metabolism, and on the reproductive- and immune systems (Encarnação et al. 2019; Gore et al. 2015; van Duursen et al. 2020; Vandenberg et al. 2012). Endocrine disruptive compounds (EDCs) can display non-monotonicity in their dose-response curves and induce effects at very low concentrations (Encarnação et al. 2019; Vandenberg et al. 2012). Sensitive toxicological assessment of POPs that are suspected, or already known, to be EDCs at human relevant concentrations is therefore critical for protecting long-term health in the population.

To date, many chemicals remain untested due to lack of regulatory requirements, limited resources, and inadequate tools for efficiently investigating relevant toxicological endpoints. While the ability to disturb steroidogenesis has been well-studied for several PFAS and selected PCBs (Behr et al. 2018; Du et al. 2013a, 2013b; Kang et al. 2016; Kraugerud et al. 2011, 2010; Rosenmai et al. 2016; Running et al. 2022; Tremoen et al. 2014; van den Dungen et al. 2015; Wang et al. 2015), there are still POPs with little to no mechanistic *in vitro* data on their potential steroidogenic effects. Some compounds, such as less studied PCBs, organochlorine pesticide residues and PBDEs, are often studied as defined mixtures. Examples include pesticide formulations, such as technical chlordane (Bondy et al. 2000), or aroclors, which are commercial PCB mixtures (Katsikantami et al. 2024). This may provide a more relevant setting for real-life exposure, but overlook complex multi-class mixtures found in human blood and gives no information on the effect of individual components. The Swedish Västerbotten Intervention Programme (VIP) is a large cohort, with individual data on plasma concentrations of a diverse range of 24 POPs (Donat-Vargas et al. 2018; Norberg et al. 2010). The majority of the POPs (20 out of 24) were present at concentrations up to 1 nM in blood, though some were detected at concentrations up to 100 nM (Donat-Vargas et al. 2018). Screening all of these compounds at a wide concentration range, or studying their mixtures in a comprehensive and high-throughput manner, is practically infeasible with *in vivo* rodent models. The development and use of new approach methodologies (NAMs) using non-animal-based assessment to expand our understanding of relevant toxicological endpoints at high throughput is therefore important (Karlsson 2023; Patinha Caldeira et al. 2022; Pierozan et al. 2023; Vermeulen et al. 2020).

The Organization for Economic Co-operation and Development (OECD) test guideline #456 describe the use of the NCI-H295R steroidogenesis assay to assess effects on estradiol and testosterone synthesis (OECD 2023). This *in vitro* bioassay is considered to be sensitive, reproducible, and consistent with results derived from *in vivo* models (Hecker et al. 2006). H295R cells express all key enzymes involved in steroidogenesis, and are frequently used to investigate effects on synthesis and metabolism of steroid hormones, and screening potential EDCs in 24-well plates (Duranova et al. 2022; Haggard et al. 2018). The aim of this study was to screen POPs for their potential effect on testosterone- and estradiol synthesis at a wide concentration range, focusing on concentrations relevant for human blood exposure. To accomplish this, we increased the throughput of the OECD#456 H295R steroidogenesis assay by using a 96-well microplate format, applied a new data analysis approach, and validated it for testing of 17β-estradiol (hereafter “estradiol”) and testosterone synthesis. The established protocol enabled higher throughput screening for endocrine disruptive properties of 24 POPs detected in human blood plasma samples from the Swedish VIP study (Donat-Vargas et al. 2018; Norberg et al. 2010).

## Method

### Screening environmental contaminants for effects on steroid hormone biosynthesis in H295R cells

To enable higher throughput *in vitro* screening of chemical effects on estradiol and testosterone synthesis, the OECD #456 H295R steroidogenesis assay in 24-well plates was downscaled to 96-well microplate format, while adhering to OECD guideline recommendations to the greatest possible extent. The shift to 96-well microplate increased the throughput, minimized the number of plates and cells used, and increased format compatibility with other commonly used assays. Another advantage is that the quality controls (forskolin, prochloraz, solvent- and medium controls) were integrated into the plate design, eliminating the need for the separate quality control plate recommended in the guideline protocol. The 96-well plate format allow testing of 16 different conditions in triplicate per plate compared to only 7 in the original 24-well plate protocol. To accommodate this set-up, the cytotoxicity control and the lowest of two concentrations of the positive controls (forskolin and prochloraz) described in the OECD TG#456 were omitted. This 96-well plate format yields 200 µL of medium for analysis of steroid hormones, a reduction of the original (1 mL/well) working volume. Here, ELISA was used to quantify estradiol and testosterone, but the set-up is compatible with other analytical methods and endpoints of interest (Källsten et al. 2022).

The adrenal cell line NCI-H295R was purchased from American Type Culture Collection (ATCC, CRL-2128, Manassas, VA, USA), and has been authenticated using STR testing by the same company. In brief, the cells were cultured in 1:1 Dulbecco’s Modified Eagle Medium/Nutrient Mixture F-12 (DMEM/F-12) containing L-glutamine and HEPES (Fisher Scientific, Waltham, USA), supplemented with 1% ITS + premix containing insulin, human transferrin, and selenous acid (Corning Inc., Bedford, USA) and 2.5% NuSerum (Fisher Scientific, Waltham, USA) in 75 cm^2^ T75 flasks (Sarstedt, Nümbrecht, Germany). Cells were maintained in a humified incubator at 37°C, 5% (v/v) CO_2_ and the medium was changed every two to three days. The cells were subcultured approximately once per week, when reaching 85-90% confluency, and experiments were conducted within passages 4-10 (OECD 2023). Cells were seeded at a density of 50 000 cells per well in 96-well tissue culture plates (Sarstedt, Nümbrecht, Germany), excluding the outermost wells to minimize edge effects. The following day, media was replaced with media containing the test compounds at a fixed concentration-range centered around low human-relevant exposures (1nM, 3 nM, 10 nM, 30 nM, 100 nM, 1 µM, 3 µM and 10 µM), as well as the positive controls forskolin (10 µM) and prochloraz (1 µM), and vehicle control (0.1% dimethyl sulfoxide, DMSO; CAS 67-68-5, Sigma-Aldrich, purity ≥99.9%). The complete plate design is described in Supplemental Table S1. All chemical stocks were visually inspected continuously to rule out compound precipitation prior to use. After 48h exposure, the number of cells and confluence was measured using a plate reader equipped with a bright field camera (SpectraMax® i3x MiniMax™ 300 Imaging Cytometer, Molecular Devices LLC, San José, USA) and media was collected and stored at -80°C for hormone analysis before the cell viability was assessed with the MTT assay. All tests were performed with technical triplicates, in three independent experiments at different days.

## Cell viability assay

After removing medium for hormone analysis, the [3-(4,5-dimethylthiazol-2-yl)-2,5-diphenyl tetrazolium bromide] (MTT) assay was conducted to quantify mitochondrial activity as a marker for cell viability (Ghasemi et al. 2023). Briefly, the cells were incubated with 50 µL of 0.5 mg/mL MTT (CAS 298-93-1, purity ≥97.5%, Sigma-Aldrich) in 1:1 DMEM/F-12 cell medium for 1h at 37°C. After removing the MTT solution, crystals were dissolved in 50 µL DMSO, and the plate was shaken for 5 minutes before the absorbance was measured at 570 nm using the plate reader. Cell viability was calculated as fold of solvent control after subtraction of blank control, rather than cytotoxicity control that was omitted due to space constraints in the 96-well plate format. Samples demonstrating <80% of solvent control cell viability were excluded from hormone analysis in accordance with the OECD guidelines.

## Estradiol and testosterone quantification

ELISA kits (ADI-901-008, and ADI-901-065, ENZO Life Sciences Inc, Farmingdale, USA) were used in accordance with the manufacturer’s instructions to analyze estradiol and testosterone levels in cell medium (200 µL). Positive controls for estradiol and testosterone suppression (1 µM prochloraz) and stimulation (10 µM forskolin) were included in each plate. The OECD guideline positive control acceptability criteria for each plate (≥7.5/≤0.5 and ≥1.5/≤0.5-fold increase/decrease for estradiol and testosterone respectively) was applied to confirm accuracy of the assay (Hecker et al. 2011; OECD 2023). To assess any variation of estradiol and testosterone levels in H295R cells, the hormone baseline synthesis in the solvent control was compared between each experiment. These datapoints were then grouped based on passage number or batch, to quantify potential influence on H295R steroidogenesis.

## Validation compounds and chemical test library

Prior to screening the chemical test library, the downscaled 96-well microplate assay was assessed for laboratory proficiency using six validation compounds, in accordance with OECD TG# 456 that defines the expected lowest observed effect concentration (LOEC) for each reference compound. The validation test chemicals forskolin (CAS 66575-29-9, F3917, LOT SLBZ0653, purity >98%), prochloraz (CAS 67747-09-5, 64947, LOT BCBT9975, purity 99.4 %), atrazine (CAS 1912-24-9, 90935, LOT BCCC8529, purity 98.1 %), bisphenol A (BPA; CAS 80-05-7, 239658, LOT MKCD7508, purity >99%), aminoglutethimide (AGT; CAS 125-84-8, A0496000, LOT 1.1, purity 98.3%) and human chorionic gonadotropin (HCG; CAS 9002-61-3, LOT 0000306756) were purchased from Sigma-Aldrich in powder form and dissolved in DMSO or pure water (HCG only). The chemical test library included the 24 POPs with highest median blood concentration previously measured in 775 adults participating in the VIP study in northern Sweden (Supplemental Table S2). These include substances from the chemical classes PFAS, PCBs, OCPs, and PBDEs. All compounds were individually dissolved in DMSO to 10-100 mM stock solutions depending on solubility, and stored in glass vials protected from light at ambient temperature (details described in Supplemental Table S2). Working solutions at 10 mM, 1 mM and 0.01 mM were prepared through dilution of the stock with DMSO in echo-qualified 384-well polypropylene microplates (Beckman Coulter Life Science, San José, USA) and stored in a light-protected environment at ambient temperature. For each experiment, the compounds were transferred from the 384-well echo stock plate to 96-well polypropylene microplates (Thermo Fisher Scientific Inc., Waltham, USA) using the Echo 550 Acoustic Liquid Handler (Beckman Coulter Life Sciences, San José, USA), sealed with a PlateLoc thermal microplate sealer and stored at -20℃, if not used immediately. The compounds were then diluted in cell media (≤0.1% DMSO) to the defined exposure concentrations and shaken at 500 RPM for 1h before application to the cell plate through a media change.

## Statistical analysis

The analysis of baseline synthesis of estradiol and testosterone by cell passage and cell batch was performed in Graphpad Prism (ver. 8.4.3), applying a one-way ANOVA followed by a Tukey’s multiple comparisons test to compare the mean steroid hormone level of every group against each other, with a significance level of 0.05. Data is presented as concentration of estradiol or testosterone by batch or passage of cells treated with solvent control (0.1% DMSO).

To analyze effects of compound treatment on hormone synthesis according to OECD TG#456, the concentration of estradiol and testosterone, was first standardized to the corresponding solvent control for each experiment. The fold of control was then analyzed by one-way ANOVA, followed by the Dunnett’s multiple comparison test in Graphpad Prism and R v.4.2.2 to identify conditions deviating from control, with a significance level of 0.05. According to the OECD test guidelines, this analysis was conducted separately for each individual experiment. Compounds for which two adjacent tested concentrations were significantly different compared to solvent control (>1.5 fold) in the first two independent experiments, or two out of three independent experiments, are considered to be disruptive to steroidogenesis.

To enable analysis of the average of all three independent experiments, and still consider potential batch-dependent variation in base synthesis of estradiol and testosterone, we also conducted statistical analysis with a linear mixed-effects model (LMM) followed by Dunnett’s multiple comparison, using the same significance level as for the ANOVA (0.05). A LMM assumes linearity, homoscedasticity and normality of residuals and is similar to a two-way ANOVA, which accounts for multiple factors that can affect the response, but is better at accounting for clustering, can handle continuous outcome variables and is more robust towards missing values (Bates et al. 2015; Galecki et al. 2006; Krueger and Tian 2004). This analysis was performed on log-normalized data, as it increased compatibility with the LMM and adherence to normality was unchanged after transformation. The code for the statistical calculation can be found here: https://github.com/flerpan01/POP-screening.

## Results

### Validation of the 96-well plate protocol for the OECD TG#456 steroidogenesis assay

The summarized results of the validation test using six proficiency substances are shown in Table 1, where the LOEC defined by using both the standard OECD data analysis and the LMM approach presented here is reported. All graphs are available in Supplemental Figs. S1-S4.

**Table 1:** LOEC for six proficiency substances defined in the OECD TG#456 to compare and validate the downscaled experimental protocol and statistical approaches for the H295R steroidogenesis assay

Compound	Estradiol				Testosterone			
		LOEC (µM) OECD guidelines	LOEC (µM) ANOVA + Dunnett’s	LOEC (µM) LMM + Dunnett’s		LOEC (µM) OECD guidelines	LOEC (µM) ANOVA + Dunnett’s	LOEC (µM) LMM + Dunnett’s
Forskolin	Strong inducer	≤ 0.1	0.3	0.1	Strong inducer	≤ 10	0.3	0.03
Prochloraz	Strong inhibitor	≤ 1	1	1	Strong inhibitor	≤ 0.1	0.03	0.1
Atrazine	Moderate inducer	≤ 10	3	10	Moderate inducer	≤ 100	3	0.03
Bisphenol A	Weak inducer	≤ 10	N/A*	0.03	Weak inhibitor	≤ 10	10	10
Aminoglutethimide	Moderate inhibitor	≤ 100	30	30	Moderate inhibitor	≤ 100	100	100
Human Chorionic Gonadotropin	Negative	N/A	N/A	N/A	Negative	N/A	N/A	N/A

^*^10 µM after calculating the average of three experiments

The testosterone LOEC identified by the standard OECD approach using ANOVA on the separate experiments correctly defined all six reference compounds according to the guidelines. For estradiol, the standard analysis of the data defined LOEC for four out of six reference compounds, including both inhibitors (prochloraz, AGT) and inducers (atrazine), as well as the negative reference compound HCG, in accordance with the OECD guideline thresholds. One exception was the strong inducer forskolin, which had a LOEC of 0.3 µM, slightly above the threshold of ≤0.1 µM. The second deviation from OECD-defined LOEC was observed in cells treated with the weak inducer BPA, where a LOEC could not be determined. However, analyzing the average estradiol concentration across the three experiments, as is common in most *in vitro* cell-based assays, resulted in a LOEC of 10 µM. Our aim was to screen the test compounds using the average of three independent experiments to consider all three biological replicates in a single analysis, thereby deviating from the OECD guidelines. This was achieved by applying the LMM to the same data. The validation was therefore also performed using the LMM as the statistical method, which defined LOECs at- or below the OECD threshold for all reference compounds in both the testosterone and estradiol measurements (Table 1). The alternative data analysis method defined LOECs that were equal (six), slightly lower (four), or higher (two) compared to the statistical method in the OECD guidelines, demonstrating similar or slightly higher sensitivity.

**Fig. 1:**
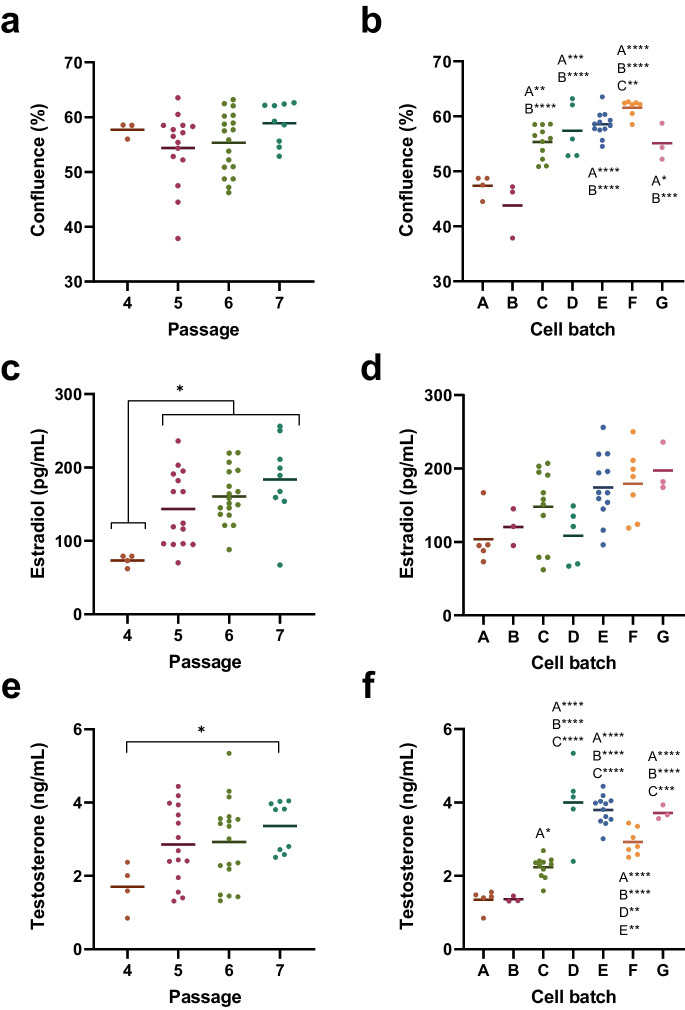
Passage-dependent and batch-dependent baseline in H295R-cells treated with solvent control (0.1% DMSO), measured by cell proliferation by cell confluence (%) (**a**,** b**), estradiol synthesis (pg/mL) (**c**,** d**) and testosterone synthesis (ng/mL (**e**,** f**). Cells were treated with 0.1% DMSO for 48h, and each datapoint represents the steroid concentration from one independent experiment, averaged from three technical replicates. Horizontal bars indicate the mean for each group. Statistical analysis was performed using ANOVA and Tukey’s multiple comparisons test. Significant differences between groups are indicated by corresponding uppercase letter and number of stars quantifying the degree of significance (e.g. A is significantly different compared to batch “A”, (*) p<0.05, (**) p<0.01, (***) p<0.001, (****) p<0.0001)

Significant batch-effects in cell proliferation and baseline steroidogenesis in the control samples were observed for both estradiol and testosterone. Mean proliferation in control, measured by % of well area covered by cells (confluence) through image analysis, was independent of passage number (Fig. 1a) but differed significantly between batches of cells (Fig. 1b). Cell viability measured by MTT, on the other hand, did not change to a significant extent between batches or cell passages. Estradiol synthesis was significantly lower in cells in passage 4 compared to passages 5-7 (Fig. 1c), but did not significantly differ by cell batch (Fig. 1d). Testosterone synthesis was also affected by passage number, although a statistically significant difference was only observed in passage 4 compared to 7 (Fig. 1e). In addition, the cell batches significantly affected testosterone synthesis (Fig. 1f).

## Compound screening in H295R cells

**Fig. 2:**
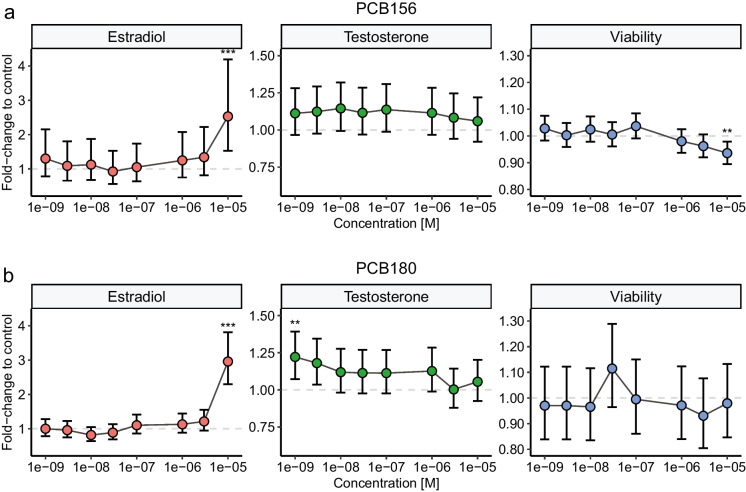
Effects of exposure to PCB-156 (**a**) or PCB-180 (**b**) on estradiol- and testosterone synthesis and viability in H295R cells treated for 48h in 96-well plates. Values represent mean ± 95% confidence interval (CI) from three independent experiments (with technical triplicates for each condition). Statistically significant differences from control are indicated as follows: *p<0.05; **p<0.01; ***p<0.001 (LMM followed by Dunnett’s multiple comparison test)

The concentration-response effects of all 24 individual POPs (eight concentrations in the range 1 nM - 10 µM) on estradiol and testosterone levels, as well as H295R cell viability, are shown in Supplemental Figs. S5-S10. According to the revised OECD criteria from 2022, none of the tested compounds were positive for effects on steroidogenesis, due to lower effect sizes than the 1.5 fold of control threshold or the requirement for significant effects at two consecutive tested concentrations (OECD 2023). Two compounds, PCB-156 and PCB-180, induced a statistically significant >1.5-fold increase in estradiol synthesis at the highest concentration tested (Fig. 2a and b). However, as this effect was observed at only one condition the OECD guideline interpretation is equivocal. Higher concentrations of the compounds may also induce these effects, but due to solubility of PCB-156 and PCB-180 in DMSO, and limitations of DMSO concentrations in cell medium, 10 µM was the highest concentration tested in this study.

**Fig. 3:**
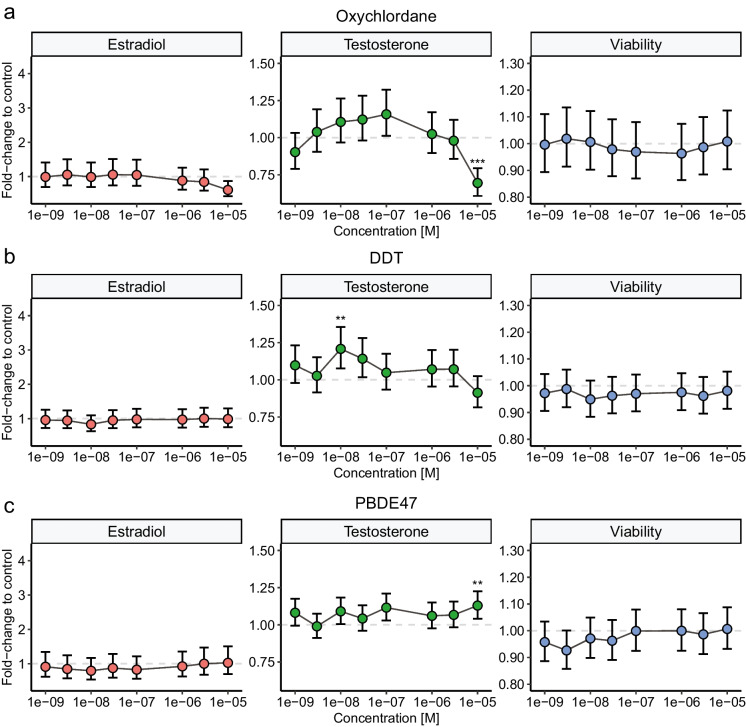
Effects of exposure to DDT (**a**), oxychlordane (**b**) or PBDE-47 (**c**) on estradiol- and testosterone synthesis and viability in H295R cells treated for 48h in 96-well plates. Values represent mean ± 95% confidence interval (CI) from three independent experiments (with technical triplicates for each condition). Statistically significant differences from control are indicated as follows: *p<0.05; **p<0.01; ***p<0.001 (LMM followed by Dunnett’s multiple comparison test)

The LOEC defined by the alternative data analysis with LMM followed by Dunnett’s multiple comparison test are presented in Table 2. Three of the tested substances, all from different chemical classes, induced small but significant effects on H295R cell viability. The viability was significantly increased by 12% after exposure to 1 nM PFUdA, while treatment with 10 µM PCB-156 led to a decrease by 6%. The viability of cells treated with PBDE-153 was also decreased compared to solvent control, by 9% at 3 nM and 1 µM, 3 µM, and 10 µM. All PFAS except PFOS and PFHxS significantly altered steroid hormone synthesis (Table 2), although the effect did not exceed the OECD criteria 1.5-fold of control. Treatment with 10 µM PFOA significantly decreased the testosterone levels by 18%. Estradiol synthesis was increased with statistical significance after treatment with PFNA (10 µM, 39%), PFDA (10 nM, 54%; 1 µM, 59%; 10 µM, 55%) and PFUdA (10 µM, 41%). Out of the ten PCBs tested here, seven compounds significantly affected testosterone and/or estradiol synthesis, though only two reached the OECD threshold of 1.5 (Table 2). Interestingly, five PCB congeners (PCB-74, PCB-99, PCB-118, PCB-138, and PCB-180) increased the testosterone levels by 16-29% at 1 nM, which was the lowest concentration tested. For both PCB-74 and PCB-180, this low-exposure effect was the only significantly affected condition, whereas PCB-99, PCB-118 and PCB-138 also induced effects at higher concentration(s). The three congeners PCB-153, PCB-156, and PCB-180 increased the estradiol levels by 40%, 153%, and 196% respectively, but only at the highest concentration 10 µM (Fig. 2). PCB-180 was the only POP to elicit effects on both testosterone and estradiol synthesis among all 24 tested compounds (Fig. 2b). Its effect on testosterone synthesis was observed specifically at 1 nM, and the magnitude of this increase (22%) was smaller compared to the increase in estradiol levels induced at 10 µM (196%). The effects of five OCPs on steroid hormone synthesis were also examined (Table 2). Overall there was no consistent pattern in the steroidogenic response of these compounds. DDE exposure induced no significant changes in the measured parameters, however, cells treated with 10 nM DDT synthesized 21% more testosterone compared to control, although this effect was not observed at other tested concentrations (Fig. 3b). Trans-nonachlor also induced elevated testosterone levels by 16% at 10 nM and 30 nM, but not at the higher concentrations. Exposure to β-HCH had no statistically significant effect on any tested endpoint. Oxychlordane, a metabolite of technical chlordane, significantly reduced testosterone levels by 30% at the highest tested concentration, 10 µM (Fig. 3b). The three PBDEs included in this screening; PBDE-47, PBDE-99, and PBDE-153, all increased testosterone synthesized in the H295R cells, in the range of 13-21% (Table 2). While PBDE-47 only elicited this effect at the highest concentration (Fig. 3c), both PBDE-99 and PBDE-153 stimulated testosterone synthesis from 10 nM and 1 nM respectively, but not in the higher concentration ranges.

**Table 2:** LOEC for cell viability, and disruption of estradiol and testosterone secretion

Compound	Viability	Estrogen	Testosterone
PFOA	-	-	10 µM, (0.82)
PFNA	-	10 µM, (1.39)	-
PFDA	-	10 nM, (1.54)	-
PFOS	-	-	-
PFHxS	-	-	-
PFUdA	1 nM, (1.12)	10 µM, (1.41)	-
PCB-74	-	-	1 nM, (1.18)
PCB-99	-	-	1 nM, (1.29)
PCB-118	-	-	1 nM, (1.16)
PCB-138	-	-	1 nM, (1.19)
PCB-153	-	10 µM, (1.40)	-
PCB-156	10 µM, (0.94)	10 µM, (2.53)	-
PCB-170	-	-	-
PCB-180	-	10 µM, (2.96)	1 nM, (1.22)
PCB-183	-	-	-
PCB-187	-	-	-
β-HCH	-	-	-
Transnonachlor	-	-	10 nM, (1.16)
Oxychlordane	-	-	10 µM, (0.70)
DDT	-	-	10 nM, (1.21)
DDE	-	-	-
PBDE-47	-	-	10 µM, (1.13)
PBDE-99	-	-	10 nM, (1.18)
PBDE-153	3 nM, (0.91)	-	1 nM, (1.21)

Lowest observed effect concentration (LOEC) of POPs in H295R cells tested at concentrations between 1 nM to 10 µM. LOEC is presented in nM or µM, and corresponding fold-change compared to solvent control (0.1% DMSO) is reported in parenthesis. Data represents mean of three independent experiments with technical triplicates (n=3). LMM followed by Dunnett’s’ multiple comparison test was used for statistical comparison with control. LOEC was identified as the lowest concentration that was significantly different compared to control

## Discussion

Disruption of the endocrine system can result in impairment of developmental processes, brain function, metabolism, and the reproductive- and immune systems (Encarnação et al. 2019; Gore et al. 2015; van Duursen et al. 2020; Vandenberg et al. 2012). It is therefore important to screen chemicals for their potential endocrine disturbing properties at human relevant concentrations. In this study an downscaled 96-well plate protocol for the OECD TG#456 steroidogenesis assay was established to screen 24 POPs detected in human blood. The results showed that six compounds significantly altered the estradiol synthesis, whereas 12 compounds affected the testosterone synthesis in the H295R cell line, although the effect size was below the OECD threshold of 1.5-fold change compared to control for all but three compounds. Three of the POPs also induced small but statistically significant effects on cell viability as measured by the MTT assay.

While the OECD guideline recommend data analysis by ANOVA on a per-experiment approach is well-established, this approach has some disadvantages. The standard practice in most *in vitro* cell-based models is to perform the statistical test on the average of minimum three independent experiments, and after standardization of the solvent control to 1, the variation between groups cannot be assumed to be equal. Strictly speaking, the ANOVA also assumes independence between groups which is broken by application of fold-change of solvent control. Additionally, this approach makes it more difficult to compare magnitude of effects between studies, as there is a lack of summarized conclusion of all independent experiments. Furthermore, according to the guidelines, only two experiments are required given that the results are equivalent in both biological replicates. Capturing the extent of biological variation between experiments is difficult with such few biological replicates. Since passage number and cell batch evidently have significant impact on baseline synthesis of estradiol and testosterone, this is particularly true for the H295R steroidogenesis assay. In this study we therefore introduced the LMM approach as an alternative that enable analysis of multiple independent experiments while considering potential batch-dependent variation. The data was log-transformed to adhere to normal distribution, and the LMM analysis was followed by Dunnett’s multiple comparison test using the lme4 package in R to identify statistically significant conditions (Bates et al. 2015). The LMM includes factors that account for random effects and uncertainty, similar to a two-way ANOVA that also considers random effects. The difference lies in the robustness, as the two-way ANOVA, like the one-way, is sensitive to missing values which the LMM is more lenient towards.

Stakeholders from the industry have expressed concern regarding the OECD H295R assay, questioning the frequency of significant but small effect size responses (Tinwell et al. 2023). The argument is that false positives may lead to unnecessary animal testing, which is contradicting to the aim of the 3Rs. The inter-laboratory validation report by Hecker et al., on the other hand, show a very low number of false positives (Hecker et al. 2011). They did however implement a 1.5-fold change threshold below which significant results would be disregarded, which was later integrated into the final OECD guideline (Hecker et al. 2011; OECD 2023; Tinwell et al. 2023). Nevertheless, it is important to know if compounds have the potential to disrupt steroid hormone synthesis, even if the effect size is small. The endocrine system is reliant on low concentrations of circulating sex steroid hormones, and their disruption could result in severe health effects (Encarnação et al. 2019; Gore et al. 2015; van Duursen et al. 2020; Vandenberg et al. 2012). Furthermore, as all 24 tested POPs were selected based on their abundance in human blood, their cumulative mixture effect may be of concern even if the effect of each individual substance is small.

The majority of *in vitro* studies using the H295R model do not report validation or testing of reference compounds, except for the positive controls prochloraz and forskolin at fixed concentrations as part of the experimental quality control. Here, we tested six reference compounds recommended by the OECD to compare the original set-up to the downscaled screening format applied to allow higher throughput. Both the standard per-experiment analysis of the fold-change using ANOVA and Dunnett’s multiple correction test, and the LMM with Dunnett’s multiple correction test were performed. The standard method correctly identified all six reference compounds when defining LOEC for testosterone synthesis. For estradiol, four out of six compounds were below the LOEC recommended by the guidelines using the same statistical method. The two exceptions were the strong inducer forskolin, and the weak inducer BPA. For forskolin, the LOEC detected using the OECD-standard ANOVA (0.3 µM) was only slightly higher compared to the OECD threshold (0.1 µM). Estradiol-synthesis in response to BPA, a weak inducer, also failed to meet the OECD criteria for LOEC on a per-experiment basis. While an ANOVA applied to the average baseline production of estradiol from three experiments defined LOEC as 10 µM, which would fulfill the criteria, two out of the three independent experiments failed to identify a LOEC. However, for the third inducer (atrazine, moderate inducer) LOEC was defined in accordance with the criteria. The LMM, on the other hand, correctly identified all reference compounds for both testosterone and estradiol. As this was the intended statistical method for this study, allowing analysis of multiple independent experiments, the assay was considered validated for the purposes of this test compound screening.

The 24 assessed POPs were included in the chemical test library based on their abundance in human blood (Donat-Vargas et al. 2018). The eight concentrations tested in this study range between 1 nM and 10 µM, focusing on low human-relevant exposures. For 20 of the 24 tested compounds, at least one person of the participants in the VIP study had a blood concentration of 1 nM or more. The only exceptions were PCB-74, oxychlordane, PBDE-99 and PBDE-153, for which the maximum detected concentration was below 1 nM. The lowest tested concentration in this study is therefore highly relevant for human exposure (Donat-Vargas et al. 2018). The highest tested concentration, 10 µM, was selected to draw parallels to previous studies, where higher concentration than detected in human samples are more frequently tested. The solubility of compounds in DMSO and threshold for DMSO content in media (0.1%) were limiting factors for determining the test range. The Echo 550 acoustic liquid dispenser that was used to generate the treatments at correct concentrations is compatible with stocks solved in pure water, or DMSO. Due to the lipophilic nature of many test compounds, we selected DMSO as a solvent to maintain equal handling strategies for the treatments. Pilot studies using elevated DMSO concentrations above the 0.1% threshold resulted in negative effects on cell adherence to the plate. Here, the nominal concentrations applied were also assumed to be the concentration at which the cells were exposed. This is standard practice in most *in vitro*-based studies, including the OECD guidelines. However, while bioavailability in the test system differs from factors that are relevant in human exposure, it still remains a variable that may impact effective concentrations in the cells. The chemical of interest might not have reached the cells to the expected extent, by instead adhering to plastics used in the test system, or media constituents. Actual exposure concentrations may therefore be lower than nominal test concentration depending on the physiochemical properties of the compound and materials used (Fischer et al. 2017; Stadnicka-Michalak et al. 2014).

By the OECD guideline definition, none of the tested PFAS, organochlorine pesticides or PBDEs would qualify as positive for endocrine disruption. The induction or inhibition of steroid hormones was too small (below 1.5-fold change compared to control), and/or the effect was only significant at non-consecutive test concentration(s). Among all tested compounds, only PCB congeners -156 and -180 and PFDA fulfill the first criteria of the OECD-defined data analysis approach, as there was >1.5-fold increase of the estradiol production at 10 µM (PCB-156, PCB-180) or 10 nM (PFDA). However, as this effect was not observed in consecutive concentrations, the conclusion of steroidogenic effects of PCB-156, PCB-180 and PFDA remains equivocal. To fulfill the OECD criteria, additional experiments that include concentrations above 10 µM are needed to confirm whether this observation is part of a dose response curve that expands beyond the range tested in this study. In cells treated with PCB-156, a small but statistically significant decrease in viability was observed at the highest concentration, which also increased estradiol synthesis. An increase in viability would motivate further questions on whether the elevation of estradiol was a product of increased proliferation, rather than a steroidogenic mechanism of action. Now, correction for the loss in viability would instead increase the fold-change in estradiol compared to control, from 2.53 to 2.7-fold.

Chemicals can affect steroid hormone levels in several ways by targeting specific key enzymes and pathways involved in their synthesis. Some chemicals may induce testosterone production, while others decrease it, by for example influencing the expression or activity of CYP17A1 and 3β-HSD. Similarly, estrogen levels can be specifically affected through mechanisms involving aromatase (CYP19A1), which converts androgens to estrogens. The results of this study demonstrated statistically significant increases in testosterone synthesis at low concentrations in the nanomolar range for PCB-74, PCB-99, PCB-118, PCB-138, PCB-180, trans-nonachlor, PBDE-99 and PBDE-153. The majority of these effects occurred at low- but not high concentrations, with only two out of twelve compounds demonstrating LOECs in the µM range. In contrast, estradiol synthesis was significantly altered at the highest concentration (10 µM) for five compounds, while only PFNA had a statistically significant LOEC in the nanomolar range. With the lack of concentration response and only singular statistically significant findings for some test compounds, it is not possible to fully rule out artefacts as potential explanations for the findings. However, as many endocrine disruptive compounds display low-concentration effects and non-monotonic dose-response (Vandenberg et al. 2012), it is also not unlikely that the effects are observed exclusively at the lowest tested concentration. The lack of response at higher test concentrations could be attributed to compensatory protective mechanisms by the cell, although additional investigation is needed to clarify this.

Our screening demonstrated that 10 µM PFOA induced a small significant decrease in testosterone synthesis. The highest concentration of PFOA in blood in the VIP cohort was 26.57 nM, though individuals with over 48 nM (20 ng/mL) have been reported in other studies (Emmett et al. 2006). The benchmark dose level, estimated at 0.3 ng/mL or 0.73 nM in serum, is based on immunotoxicity in children, rather than disruption of steroidogenesis (Stockholm Convention on Persistent Organic Pollutants 2016). Exposure to PFOA is reported to inhibit testicular testosterone biosynthesis and decrease serum testosterone, and has been as a potential mechanism of action in the formation of Leydig cell tumors (U.S. Environmental Protection Agency 2024). Previous investigations on the effects of PFOA on steroidogenesis in H295R cells have reported similar decreases in testosterone synthesis after exposure to 30, 100 and 300 nM (Du et al. 2013b) and 100 µM (Kang et al. 2016). Other studies contradict these findings and report no significant effects of PFOA on testosterone synthesis after exposure to PFOA at 1 µM (Running et al. 2022), 1.6 µM (Rosenmai et al. 2016) and 100 µM (Behr et al. 2018; Wang et al. 2015). Another study reported moderately increased testosterone production at 600 nM and 6 µM (Kraugerud et al. 2011), further contradicting the above mentioned studies. While most studies make some efforts to adhere to the OECD test guidelines, there are differences in the experimental procedure that could motivate the observed difference in effects. The steroidogenic enzyme encoded by *CYP11A*, which is crucial in the conversion of cholesterol into pregnenolone and act as the first rate-limiting step in steroidogenesis, was downregulated in two studies (Du et al. 2013b; Kraugerud et al. 2011). This could play a part in the observed decrease in testosterone synthesis, although our and their studies did not observe a decrease in estradiol levels. Compensatory activity within the steroidogenic pathway might have maintained consistent estradiol synthesis, possibly through an increase in aromatase gene expression (Du et al. 2013b) and activity (Kraugerud et al. 2011). Additional investigation into expression- and activity levels of steroidogenic enzymes would provide additional information on the mechanism of action of PFOA, and other POPs on H295R synthesis of testosterone and estradiol.

With the large number of unique PCB congeners in technical mixtures and the environment, many have not yet been investigated for steroidogenic effects using the H295R assay. Although PCB-180 is commonly detected in human blood, it is poorly studied compared to other congeners. One previous *in vitro* study found similar positive effects on estradiol levels as shown here when exposing bovine granulosa cells to 7.6 µM of PCB-180 (Mlynarcikova et al. 2014). In our study, PCB-153 induced estradiol synthesis, while no significant effects on testosterone or cell viability were noted. Other studies of PCB-153 in H295R cells have reported similar effects on estradiol, with significantly increased synthesis at 3 µM, 4 µM and 12.7 µM (Kraugerud et al. 2010; Tremoen et al. 2014; van den Dungen et al. 2015). This indicates that the compound affects targets more downstream in the steroid synthesis pathways. Oxychlordane was one of only two tested POPs that reduced testosterone synthesis. This effect was not biased by any effects on cell viability, and no corresponding decrease in estradiol was noted. Oxychlordane has not been commonly studied for its toxicological potential as it is a metabolite of the component chlordane in the pesticide mixture technical chlordane. In a study where female rats were treated with the mixture technical chlordane, lower plasma levels of testosterone compared to controls was noted (Cassidy et al. 1994), which is consistent with our findings. All three tested PBDE congeners induced a small (13-21%), but significant increase of testosterone synthesis in H295R cells. For PBDE-153 and PBDE-99 this effect was observed at concentrations as low as 1 nM and 10 nM, respectively. Similar effects at low concentration have previously been reported in a other *in vitro* cell model, using ovarian follicles, which synthesized increased levels of testosterone after exposure to 1 nM PBDE-47 or 0.44 nM PBDE-99 for 24h (Karpeta et al. 2011). Neither treatment elicited any effects on estradiol synthesis, which is similar to the findings in our study. The results are, however, contradicted by studies using a co-culture of granulosa- and theca cells from pig ovarian follicles (Gregoraszczuk et al. 2008; Rak et al. 2017).

Comparing studies using H295R cells reveals clear methodological differences. Some studies stimulate baseline cellular steroidogenesis with forskolin or other compounds to increase the dynamic range of the assay, although this is not in the guidelines (Haggard et al. 2018; Källsten et al. 2022). Other studies treat cells for 24h instead of 48h (Running et al. 2022). The use of alternative plate-formats, such as 96-well plates or dishes, is also not covered by the guidelines (OECD 2023). Variations exist in the number of independent experiments, pre-incubation length, sub-culturing vessel, and maximum DMSO concentration used during treatment. Media formulations also have a clear impact on cell behavior. The cortisol levels have for example been shown to be affected by differences in culture medium (Duranova et al. 2022; Kurlbaum et al. 2020). Even minor differences in procedure may alter cell characteristics, which could explain different findings across studies (Behr et al. 2018; Duranova et al. 2022; Kurlbaum et al. 2020). The passage number is another factor that impact the H295R cellular steroidogenesis. OECD TG#456 is therefore clear that the original cell stock received from the provider should be passaged five times before freezing multiple batches of cells, and that these cells only should be used between passage 4 and 10 after thawing. These recommendations were included to reduce variation between experiments and laboratories (Duranova et al. 2022; Hecker et al. 2011; OECD 2023), but is not followed by many studies. Our study confirmed the importance of passage number, and revealed significant differences in base hormone synthesis even between passage 4 to 7. Another noteworthy finding was the significant differences in baseline testosterone synthesis between cryo-preserved batches of cells. This was initially thought to be due to increases in cell viability, but the viability of cells measured by MTT was not dependent on neither cell passage nor batch. The cell confluence in wells, however, was affected by cell batch, but not passage. An increase in covered cell area without correlating increase in cell viability could be indicative of changes in metabolism, or cell morphology, but additional experiments are required to determine this. The difference in sensitivity to batch-effect between testosterone and estradiol was also an unexpected finding. The statistical significance could be lacking due to the higher variability in the estradiol measurements, likely caused by the low concentration in medium which leads to greater fluctuations even with small changes in estradiol synthesis. True variation in estradiol synthesis by batch might thereby be masked by fluctuations caused by non-specific conditions due to technical variation. Testosterone, by contrast, is detected at higher concentration in H295R cell medium, and small changes due to non-specific treatment conditions are thus less likely to impact the base production across experiments. To our knowledge, these batch effects in cell behavior in H295R cells from the same stock has not been reported before.

The results derived from this human cell assay could be used as motivation for additional mechanistic investigations using *in vitro* techniques to further elucidate *in vivo* and human relevance. The expanded 96-well microplate format allows for multiplexing with additional plate-based assays, including high content imaging. This technique would allow screening for additional endpoints of interest, including oxidative stress and mitochondrial health, or levels of relevant proteins such as steroidogenic enzymes. The higher throughput format could also enable analysis molecular alterations by omics methods. Another advantage of the increased screening capacity includes the possibility of investigating customized mixtures of interest at higher throughput, which is also compatible with the acoustic liquid handling utilized here. Discoveries of additional affected toxicological endpoints or other indications of endocrine disruption could then warrant further studies in whole organisms.

## Conclusion

The OECD TG#456 test was downscaled for higher throughput to screen 24 POPs detected in human plasma for potential disruption of testosterone and estradiol synthesis in 96-well plates in a wide concentration range (1 nM to 10 µM). In total, 17 compounds induced statistically significant effects on testosterone or estradiol synthesis in H295R cells, using an alternative LMM statistical approach to analyze three independent experiments, while considering batch-dependent effects. Increased testosterone synthesis was demonstrated even at 1 nM for PCB-74 (18%), PCB-99 (29%), PCB-118 (16%), PCB-138 (19%), PCB-180 (22%), and PBDE-153 (21%). This shows that some POPs have the potential to interfere with endocrine signaling at concentrations found in human blood, and may be a concern to human development and health. The established 96-well screening platform could be useful for mechanistic studies of single compounds and mixtures. We recommend the presented alternative data analysis approach for similar studies in H295R cells as it effectively addresses batch-dependent variations and allows for the inclusion of multiple independent experiments, providing a more robust and comprehensive analysis. Unlike the OECD per-experiment approach based on ANOVA, our LMM approach captures biological variation better and accommodates missing values. This also allows adaption of the protocol to include measurements of other relevant endpoints of interest.

## Supplementary Information

Below is the link to the electronic supplementary material.

Supplementary file1 (DOCX 3.11 MB)

## Data Availability

All data supporting the findings of this study are available within the paper and its Supplementary Information.
