# Supplementary material for: Screening persistent organic pollutants for effects on testosterone and estrogen synthesis at human-relevant concentrations using H295R cells in 96-well plates
Source: Cell Biol Toxicol. 2024 Aug 13;40(1):69. doi: 10.1007/s10565-024-09902-4 (PMC11322491; doi:10.1007/s10565-024-09902-4)
Supplement: Supplementary file 1 — Supplementary file1 (DOCX 3.11 MB) [file 10565_2024_9902_MOESM1_ESM.docx]

**SUPPLEMENTARY INFORMATION**

**Screening persistent organic pollutants for effects on testosterone and estrogen synthesis at human-relevant concentrations using H295R cells in 96-well plates**

Denise Strand^1^, Erik Nylander^1^, Andrey Höglund^1^, Bo Lundgren^2^, Jonathan W Martin^1^, Oskar Karlsson^1^

^1^ Science for Life Laboratory, Department of Environmental Science, Stockholm University, Stockholm, 114 18, Sweden

^2^ Science for Life Laboratory, Biochemical and Cellular Assay unit, Dept. of Biochemistry and Biophysics, Stockholm University, Stockholm, 106 91, Sweden

Corresponding author: Oskar Karlsson, Science for Life Laboratory, Department of Environmental Science, Stockholm University, 11418, Stockholm, Sweden.

E-mail: [Oskar.Karlsson@aces.su.se](mailto:Oskar.Karlsson@aces.su.se)

|  | 1 | 2 | 3 | 4 | 5 | 6 | 7 | 8 | 9 | 10 | 11 | 12 |
| --- | --- | --- | --- | --- | --- | --- | --- | --- | --- | --- | --- | --- |
| A |  |  |  |  |  |  |  |  |  |  |  |  |
| B |  | DMSO (0.1%) | Cmpd 1 1 nM | Cmpd 1 3 nM | Cmpd 1 10 nM | Cmpd 1 30 nM | Cmpd 1 100 nM | Cmpd 1 1 µM | Cmpd 1 3 µM | Cmpd 1 10 µM | Prochloraz 1 µM |  |
| C |  | DMSO (0.1%) | Cmpd 1 1 nM | Cmpd 1 3 nM | Cmpd 1 10 nM | Cmpd 1 30 nM | Cmpd 1 100 nM | Cmpd 1 1 µM | Cmpd 1 3 µM | Cmpd 1 10 µM | Prochloraz 1 µM |  |
| D |  | DMSO (0.1%) | Cmpd 1 1 nM | Cmpd 1 3 nM | Cmpd 1 10 nM | Cmpd 1 30 nM | Cmpd 1 100 nM | Cmpd 1 1 µM | Cmpd 1 3 µM | Cmpd 1 10 µM | Prochloraz 1 µM |  |
| E |  | BLANK | Cmpd 2 1 nM | Cmpd 2 3 nM | Cmpd 2 10 nM | Cmpd 2 30 nM | Cmpd 2 100 nM | Cmpd 2 1 µM | Cmpd 2 3 µM | Cmpd 2 10 µM | Forskolin 10 µM |  |
| F |  | BLANK | Cmpd 2 1 nM | Cmpd 2 3 nM | Cmpd 2 10 nM | Cmpd 2 30 nM | Cmpd 2 100 nM | Cmpd 2 1 µM | Cmpd 2 3 µM | Cmpd 2 10 µM | Forskolin 10 µM |  |
| G |  | BLANK | Cmpd 2 1 nM | Cmpd 2 3 nM | Cmpd 2 10 nM | Cmpd 2 30 nM | Cmpd 2 100 nM | Cmpd 2 1 µM | Cmpd 2 3 µM | Cmpd 2 10 µM | Forskolin 10 µM |  |
| H |  |  |  |  |  |  |  |  |  |  |  |  |

***Table S1****. Layout of test chemicals in 96-well plate format for steroidogenesis screening using H295R cells.*

*Cells in DMSO (0.1%) wells are treated with the solvent (0.1% DMSO) and are used as control to which all treatments are compared. Cells in “BLANK” wells are incubated with regular, non-treated medium without solvent. Prochloraz and forskolin are included as positive controls for inhibition and induction, respectively. “Cmpd 1-2” annotate test compounds of interest, while concentrations in nM or µM used in this study are listed below.*

| **Class** | **Compound** | **Full name** | **CAS:** | **Purity**  **(%)** | **Manufacture** | **LOT number** | **Median blood concentration in VIP study (nM)** | **Maximum blood concentration in VIP study (nM)** | **Maximum stock concentration in DMSO (mM)** |
| --- | --- | --- | --- | --- | --- | --- | --- | --- | --- |
| **PFAS** | PFOA | perfluorooctanoic acid | 335-67-1 | 99 | Wellington Laboratories | PFOAGA05 | 6.28 | 26.57 | 100 |
|  | PFNA | perfluorononanoic acid | 375-95-1 | 99 | Wellington Laboratories | 01479 | 1.64 | 7.97 | 100 |
|  | PFDA | perfluorodecanoic acid | 335-76-2 | 99 | Wellington Laboratories | PFDAGA05 | 0.66 | 3.11 | 100 |
|  | PFUdA | perfluoroundecanoic acid | 2058-94-8 | 99 | Wellington Laboratories |  | 0.46 | 3.37 | 100 |
|  | PFHxS | sodium perfluorohexanesulfonate | 82382-12-5 | 99 | Wellington Laboratories | LPFHxSAM15 | 2.61 | 28.43 | 100 |
|  | PFOS | potassium perfluorooctanesulfonate | 2795-39-3 | 99 | Wellington Laboratories | LPFOSKAM14a | 29.73 | 111.48 | 100 |
| **PCB** | PCB-74 | 2,4,4',5-Tetrachlorobiphenyl | 32690-93-0 | 99 | Cambridge Isotope Laboratories | I-25669 | 0.11 | 0.73 | 20 |
|  | PCB-99 | 2,2',4,4',5-Pentachlorobiphenyl | 38380-01-7 | 99 | LGC Standards | 1025556 | 0.14 | 1.14 | 100 |
|  | PCB-118 | 2,3',4,4',5-Pentachlorobiphenyl | 31508-00-6 | 100 | LGC Standards | X10881359 | 0.30 | 3.13 | 100 |
|  | PCB-153 | 2,2',4,4',5,5'-Hexachlorbiphenyl | 35065-27-1 | 100 | LGC Standards | X19881359 | 2.07 | 16.42 | 100 |
|  | PCB-138 | 2,2',3,4,4',5'-Hexachlorbiphenyl | 35065-28-2 | 99 | LGC Standards | X10881359 | 1.33 | 10.31 | 100 |
|  | PCB-156 | 2,3,3',4,4',5-Hexachlorobiphenyl | 38380-08-4 | 99 | LGC Standards | X10881359 | 0.24 | 2.29 | 10 |
|  | PCB-187 | 2,2',3,4',5,5',6-Heptachlorbiphenyl | 52663-68-0 | 100 | LGC Standards | X10881359 | 0.41 | 4.65 | 10 |
|  | PCB-183 | 2,2',3,4,4',5',6-Heptachlorobiphenyl | 52663-69-1 | 98 | LGC Standards | X10881359 | 0.16 | 1.54 | 100 |
|  | PCB-180 | 2,2',3,4,4',5,5'-Heptachlorbiphenyl | 35065-29-3 | 98 | LGC Standards | X10881359 | 1.44 | 11.69 | 10 |
|  | PCB-170 | 2,2',3,3',4,4',5-Heptachlorobiphenyl | 35065-30-6 | 100 | LGC Standards | X10881359 | 0.69 | 5.40 | 20 |
| **OCP** | β-HCH | β-hexachlorocyclohexane | 319-85-7 | 98 | LGC Standards | G1047073 | 0.26 | 3.52 | 100 |
|  | Oxychlordane |  | 27304-13-8 | 100 | Cambridge Isotope Laboratories | I-20438 | 0.10 | 0.80 | 100 |
|  | Trans-nonachlor |  | 39765-80-5 | 99 | LGC (DRE) | G145996 | 0.13 | 1.86 | 100 |
|  | 4,4´-DDT | 1,1,1-Trichloro-2,2-bis (p-chlorophenyl) ethane | 50-29-3 | 100 | LGC (DRE) | G832442 | 0.10 | 0.89 | 100 |
|  | p,p'-DDE | 1,1-Dichloro-2,2-bis(p-chlorophenyl) ethylene | 72-55-9 | 98 | Toronto Research Chemicals |  | 3.52 | 26.39 | 100 |
| **PBDE** | PBDE-47 | 2,2',4,4'-Tetrabromodiphenyl ether | 5436-43-1 | 98 | LGC (DRE) | X10881359 | 0.05 | 1.06 | 100 |
|  | PBDE-99 | 2,2',4,4',5-Pentabromodiphenyl ether | 60348-60-9 | 100 | Cambridge Isotope Laboratories | PR-25045 | 0.03 | 0.26 | 100 |
|  | PBDE-153 | 2,2',4,4',5,5'-Hexabromodiphenyl ether | 68631-49-2 | 100 | Cambridge Isotope Laboratories | PR-23460 | 0.06 | 0.19 | 10 |

***Table S2.*** *List of the persistent environmental chemicals detected in human blood plasma used in this study*

*
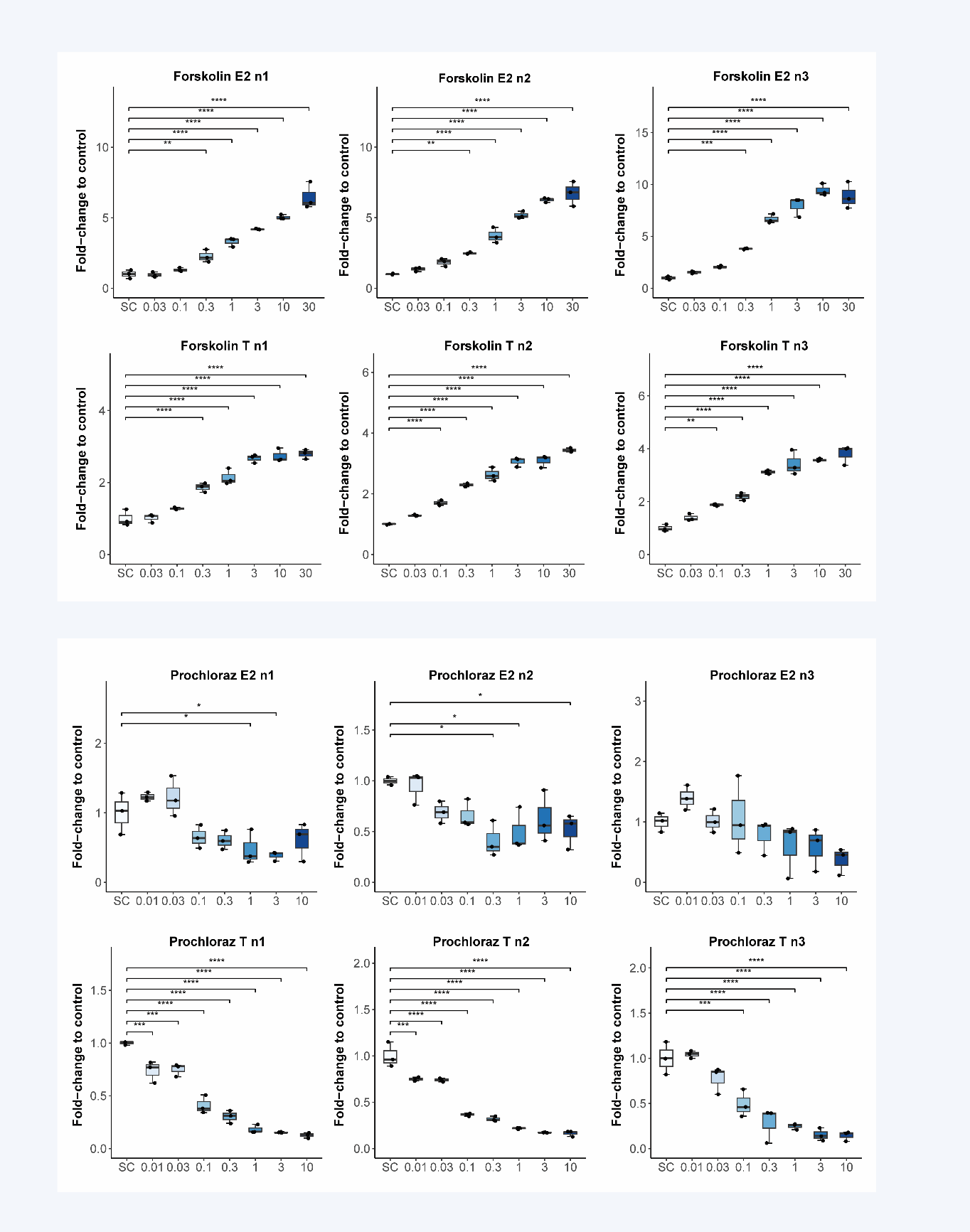
*

***Figure S1****. H295R assay test of reference compounds forskolin and prochloraz as recommended by the OECD test guidelines. Data was standardized to fold of solvent control, and each independent experiment was separately analyzed and visualized in R v.4.2.2. Stars indicate statistical significance compared to selvent control (SC) in a one-way ANOVA and Dunnet’s multiple comparison test (*p<0.05; **p<0.01; ***p<0.001, ****p<0.0001). Concentration of test compound on x-axis presented in µM*

*
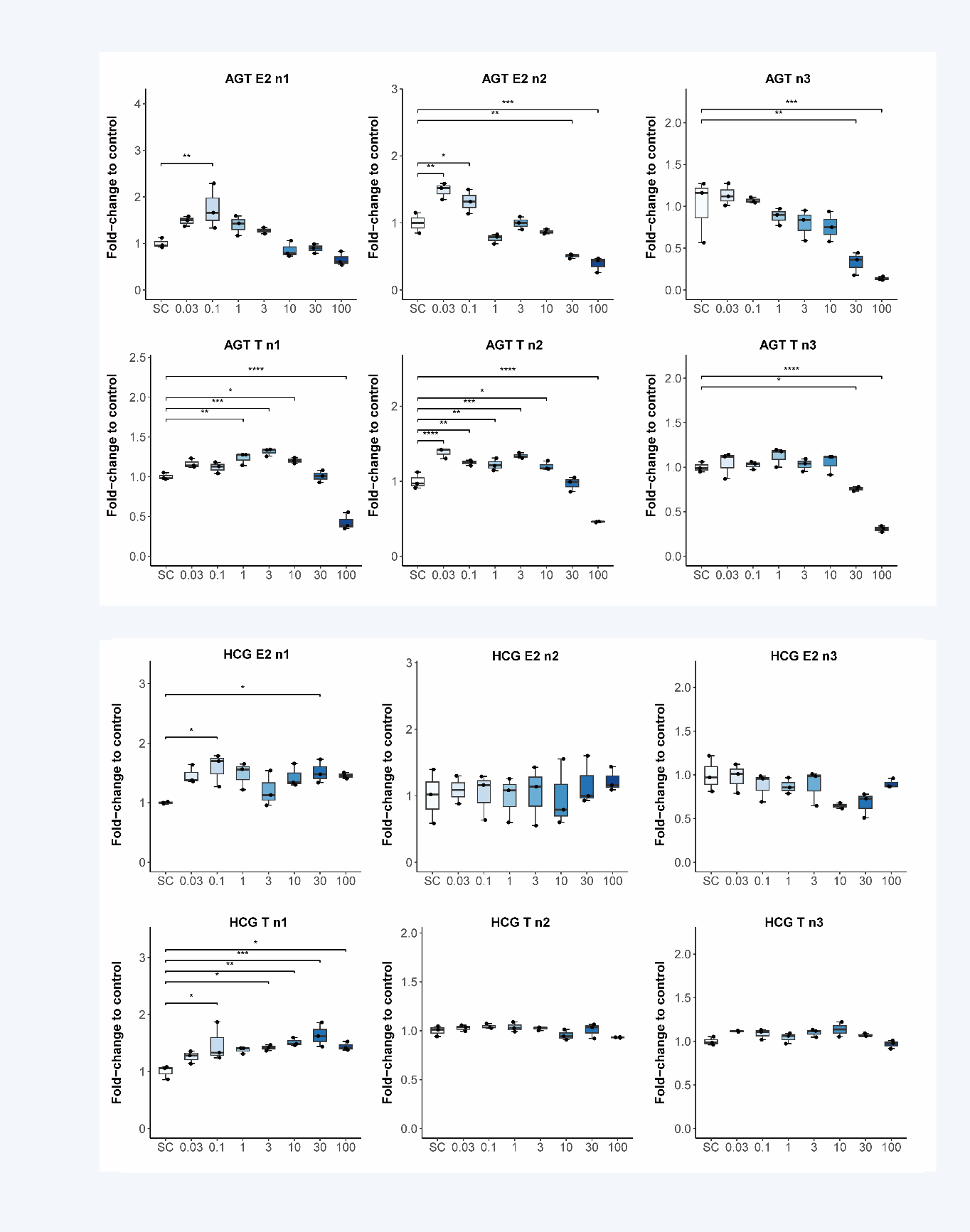
*

***Figure S2****. H295R assay test of reference compounds atrazine and Bisphenol A (BPA) as recommended by the OECD test guidelines. Data was standardized to fold of solvent control, and each independent experiment was separately analyzed and visualized in R v.4.2.2. Stars indicate statistical significance compared to selvent control (SC) in a one-way ANOVA and Dunnet’s multiple comparison test (*p<0.05; **p<0.01; ***p<0.001, ****p<0.0001). Concentration of test compound on x-axis presented in µM*

*
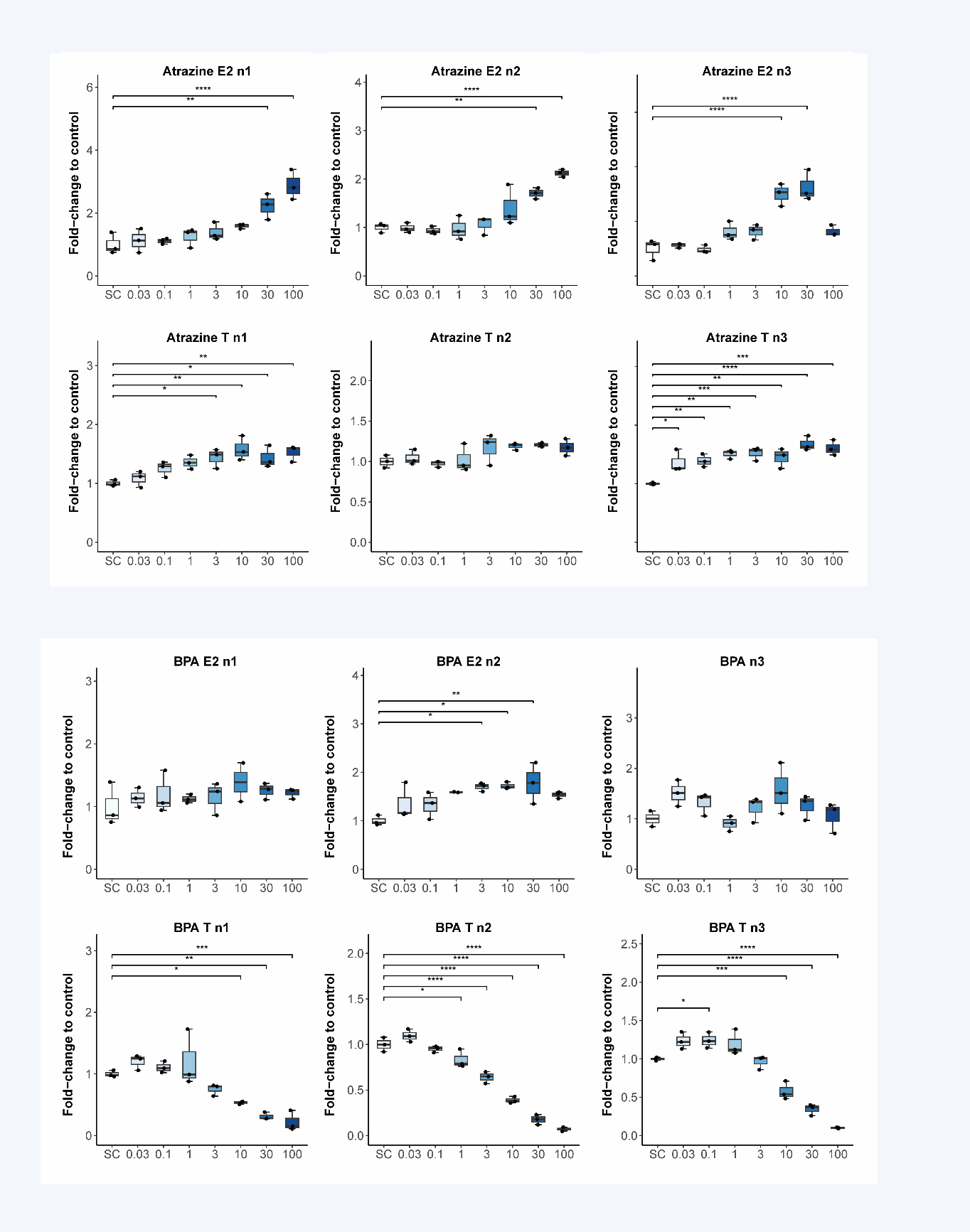
*

***Figure S3****. H295R assay test of reference compounds aminoglutethimide (AGT) and Human Chorionic Gonadotropin (HCG)as recommended by the OECD test guidelines. Data was standardized to fold of solvent control, and each independent experiment was separately analyzed and visualized in R v.4.2.2. Stars indicate statistical significance compared to selvent control (SC) in a one-way ANOVA and Dunnet’s multiple comparison test (*p<0.05; **p<0.01; ***p<0.001, ****p<0.0001). Concentration of test compound on x-axis presented in µM.
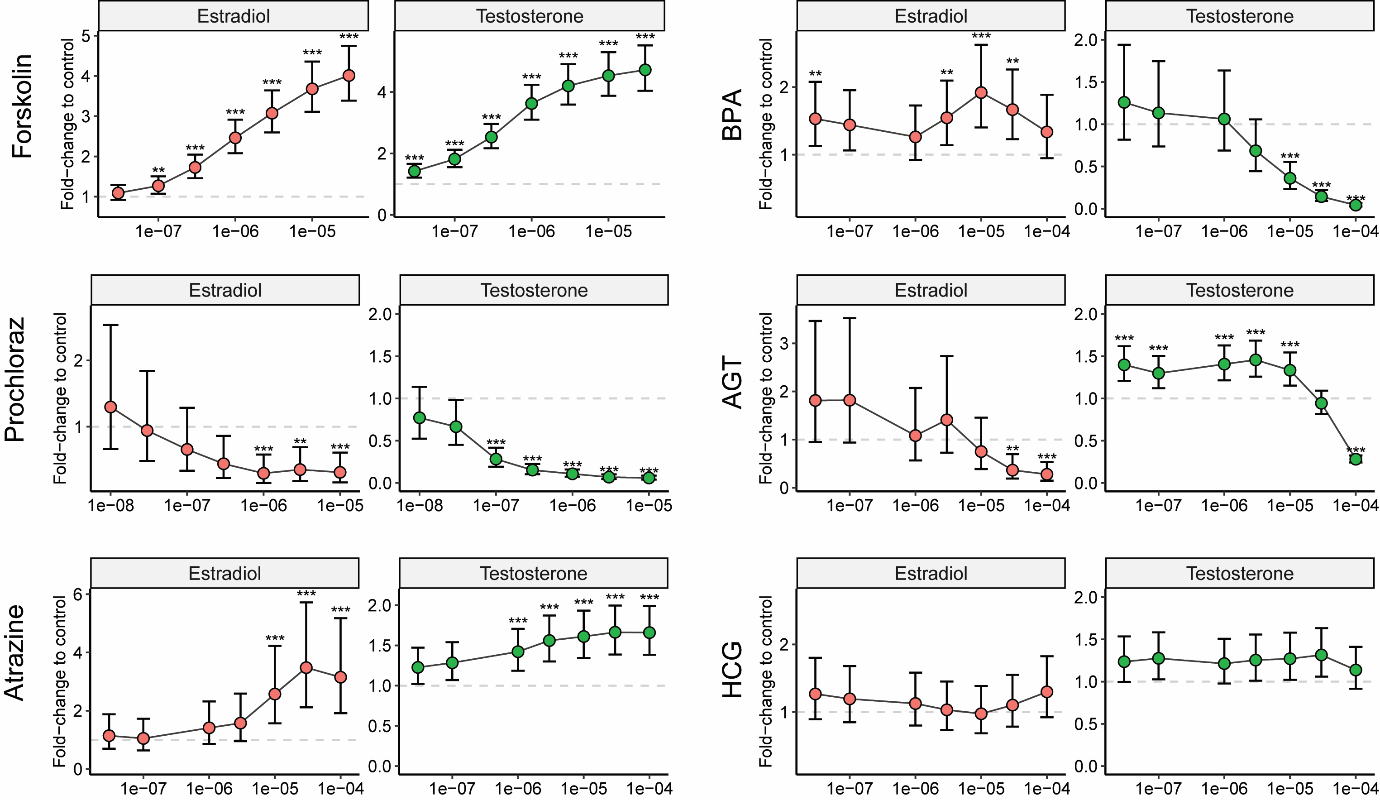
*

***Figure S4****. H295R assay test of six reference compounds recommended by the OECD test guidelines. Data was log-transformed and analysed with a LMM followed by a Dunnett’s multiple comparison test and visualized in R v.4.2.2. Values represent mean ± 95% confidence interval (CI) from three independent experiments (with technical triplicates for each condition). Statistically significant differences from control are indicated as follows: (*p<0.05; **p<0.01; ***p<0.001, ****p<0.0001). Concentrations of test compound on x-axis presented in M.*


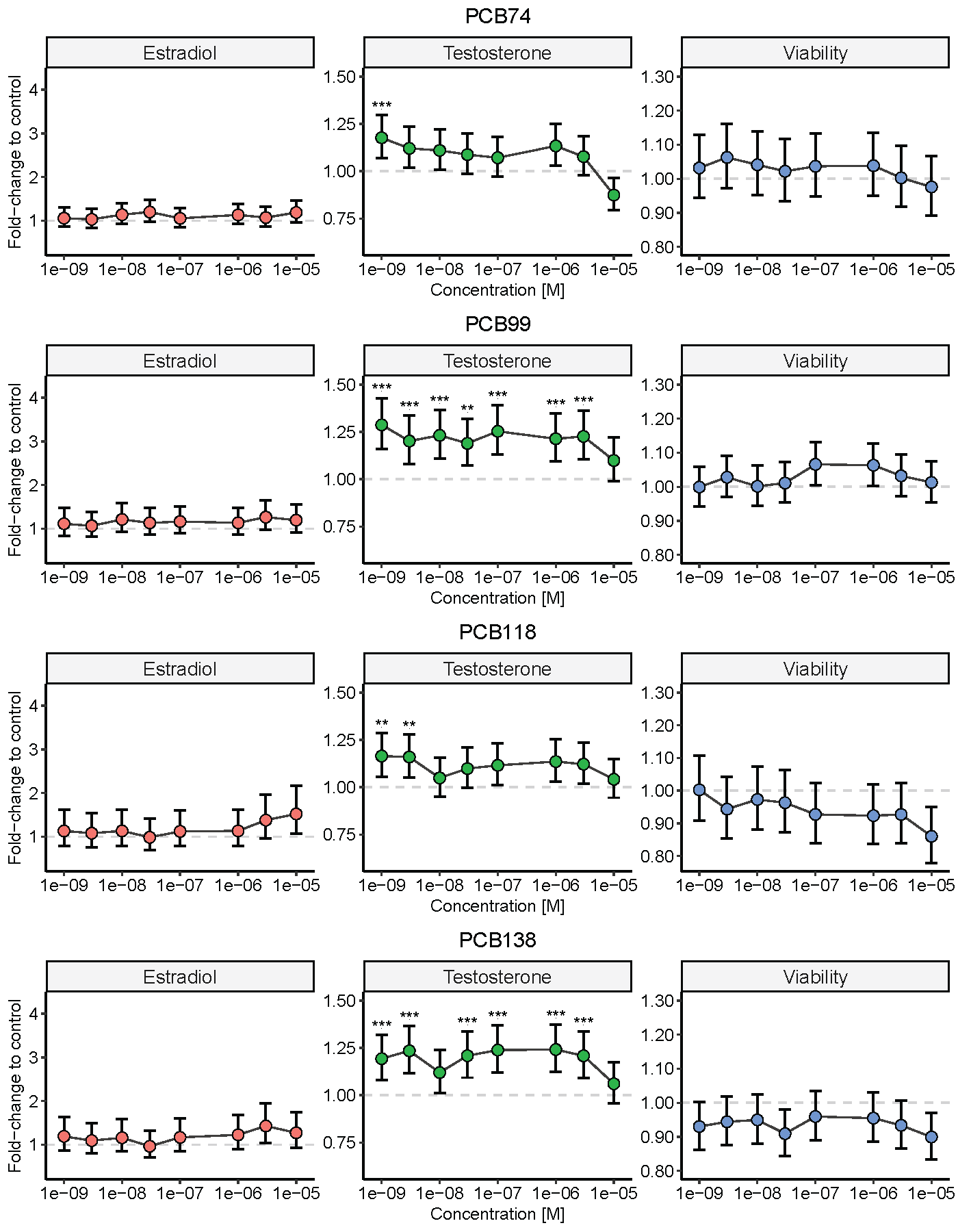


***Figure S5.*** *Screen of PCB74, PCB99, PCB118, and PCB138 for effects on estradiol synthesis (red, left graph), testosterone synthesis (green, middle graph) and viability (blue, right graph) in H295R cells treated for 48h with 8 different concentrations ranging between 1nM-10 µM. Results are presented as fold of vehicle control (0.1% DMSO). Values repredent the mean of three experiments, each in technical triplicates and plotted with ± 95% confidence interval (CI). Statistical significance was calculated by LMM + Dunnett’s’ test and indicated with stars (*p<0.05; **p<0.01; ***p<0.001).*


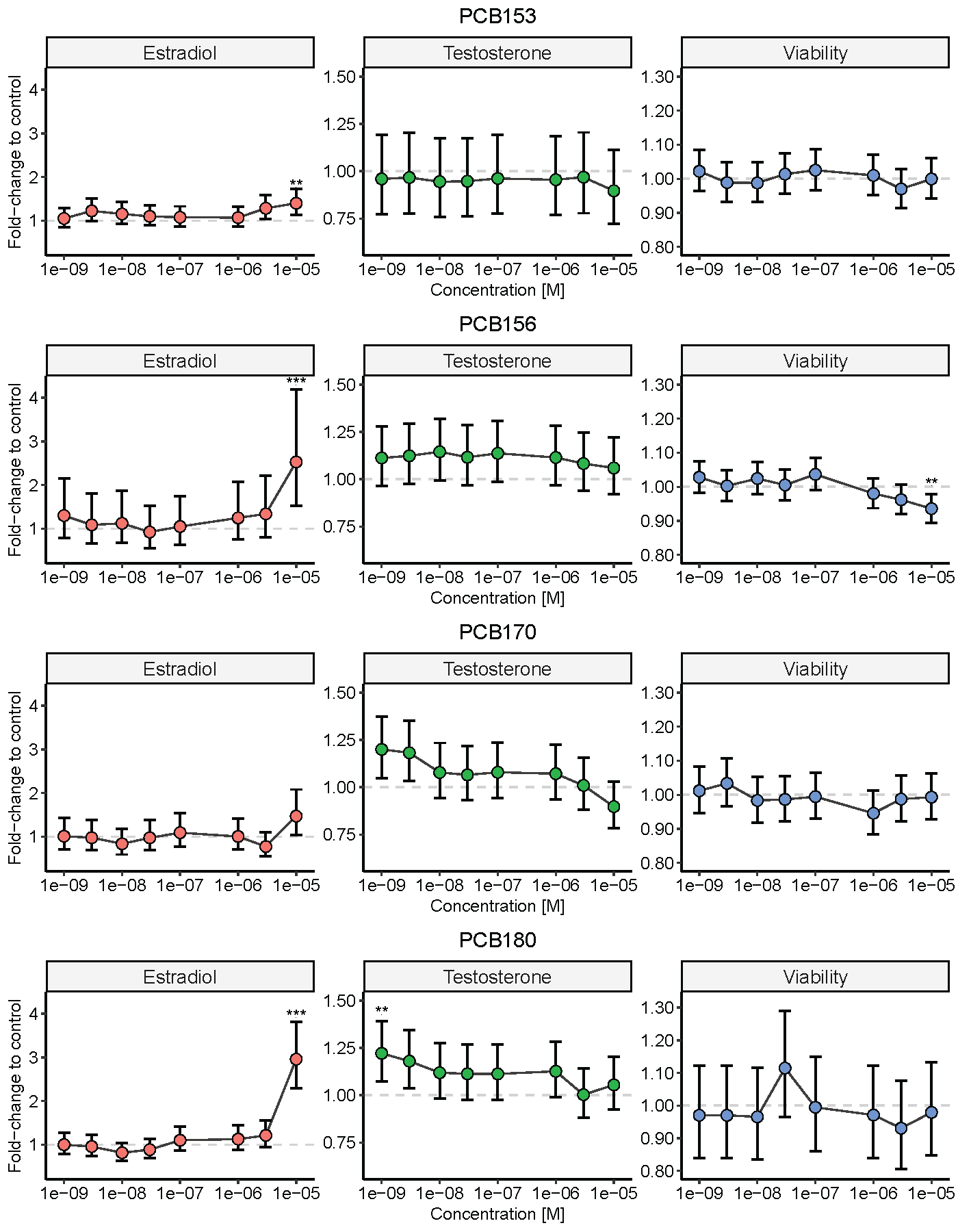
***Figure S6.*** *Screen of PCB153, PCB156, PCB170, and PCB180 for effects on estradiol synthesis (red, left graph), testosterone synthesis (green, middle graph) and viability (blue, right graph) in H295R cells treated for 48h with 8 different concentrations ranging between 1nM-10 µM. Results are presented as fold-change of vehicle control (0.1% DMSO). Values repredent the mean of three experiments, each in technical triplicates and plotted with ± 95% confidence interval (CI). Statistical significance was calculated by LMM + Dunnett’s’ test and indicated with stars (*p<0.05; **p<0.01; ***p<0.001).*


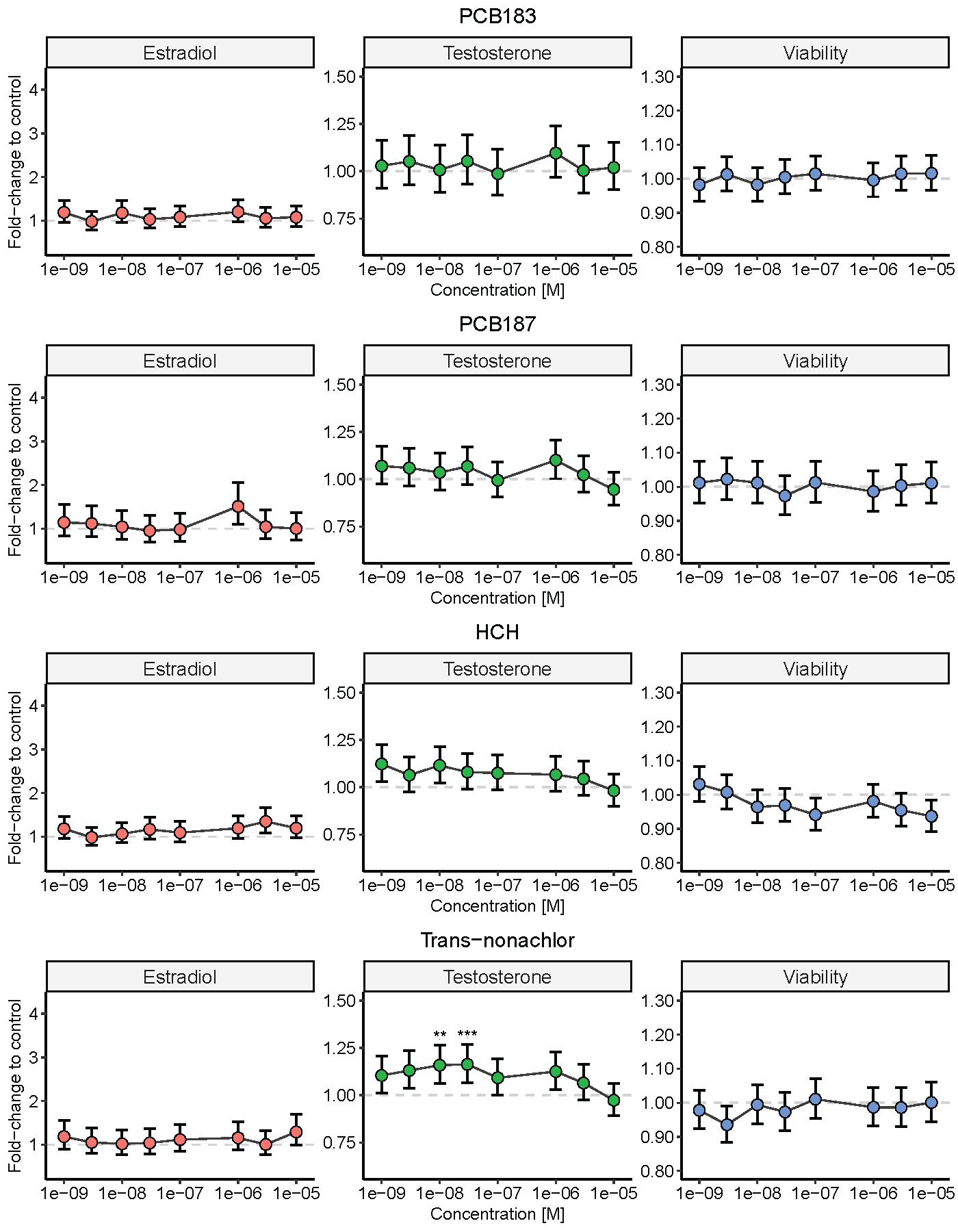


***Figure S7.*** *Screen of PCB183, PCB187, HCH, and trans-nonachlor for effects on estradiol synthesis (red, left graph), testosterone synthesis (green, middle graph) and viability (blue, right graph) in H295R cells treated for 48h with 8 different concentrations ranging between 1nM-10 µM. Results are presented as fold-change of vehicle control (0.1% DMSO). Values repredent the mean of three experiments, each in technical triplicates and plotted with ± 95% confidence interval (CI). Statistical significance was calculated by LMM + Dunnett’s’ test and indicated with stars (*p<0.05; **p<0.01; ***p<0.001).*


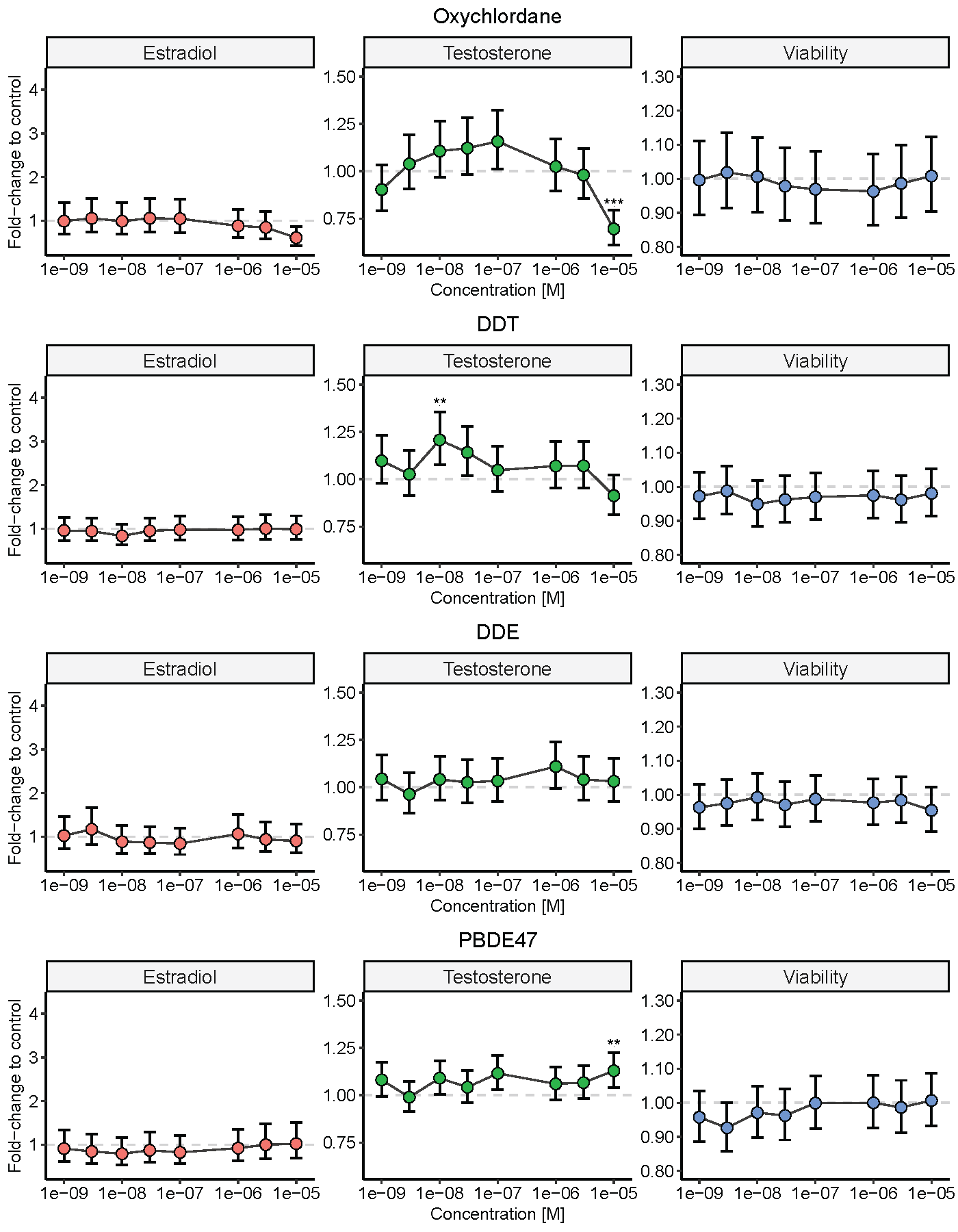


***Figure S8.*** *Screen of oxychlordane, DDT, DDE, and BDE47 for effects on estradiol synthesis (red, left graph), testosterone synthesis (green, middle graph) and viability (blue, right graph) in H295R cells treated for 48h with 8 different concentrations ranging between 1nM-10 µM. Results are presented as fold-change of vehicle control (0.1% DMSO). Values repredent the mean of three experiments, each in technical triplicates and plotted with ± 95% confidence interval (CI). Statistical significance was calculated by LMM + Dunnett’s’ test and indicated with stars (*p<0.05; **p<0.01; ***p<0.001).*


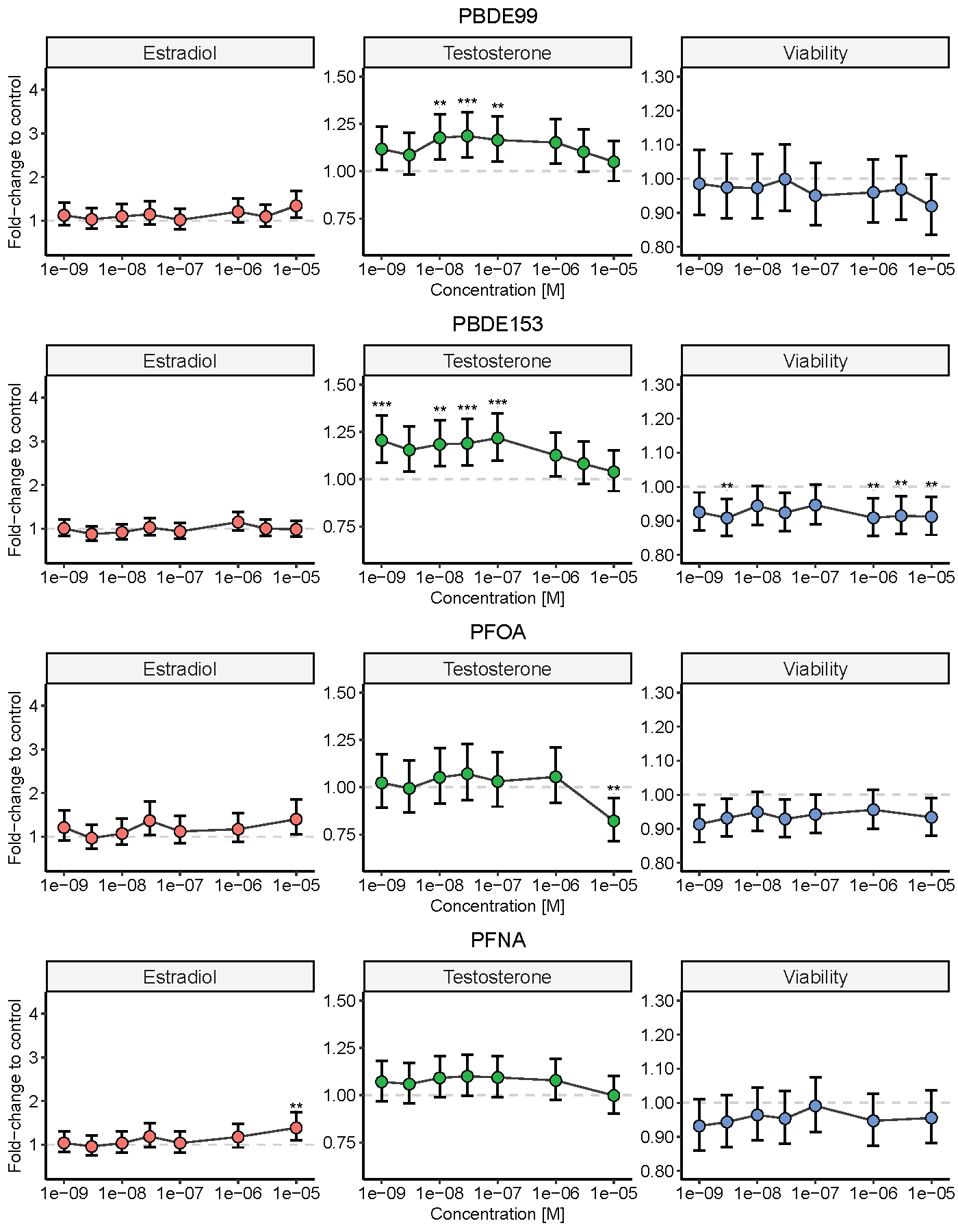


***Figure S9.*** *Screen of BDE99, BDE153, PFOA, and PFNA for effects on estradiol synthesis (red, left graph), testosterone synthesis (green, middle graph) and viability (blue, right graph) in H295R cells treated for 48h with 8 different concentrations ranging between 1nM-10 µM. Results are presented as fold-change of vehicle control (0.1% DMSO). Values repredent the mean of three experiments, each in technical triplicates and plotted with ± 95% confidence interval (CI). Statistical significance was calculated by LMM + Dunnett’s’ test and indicated with stars (*p<0.05; **p<0.01; ***p<0.001).*


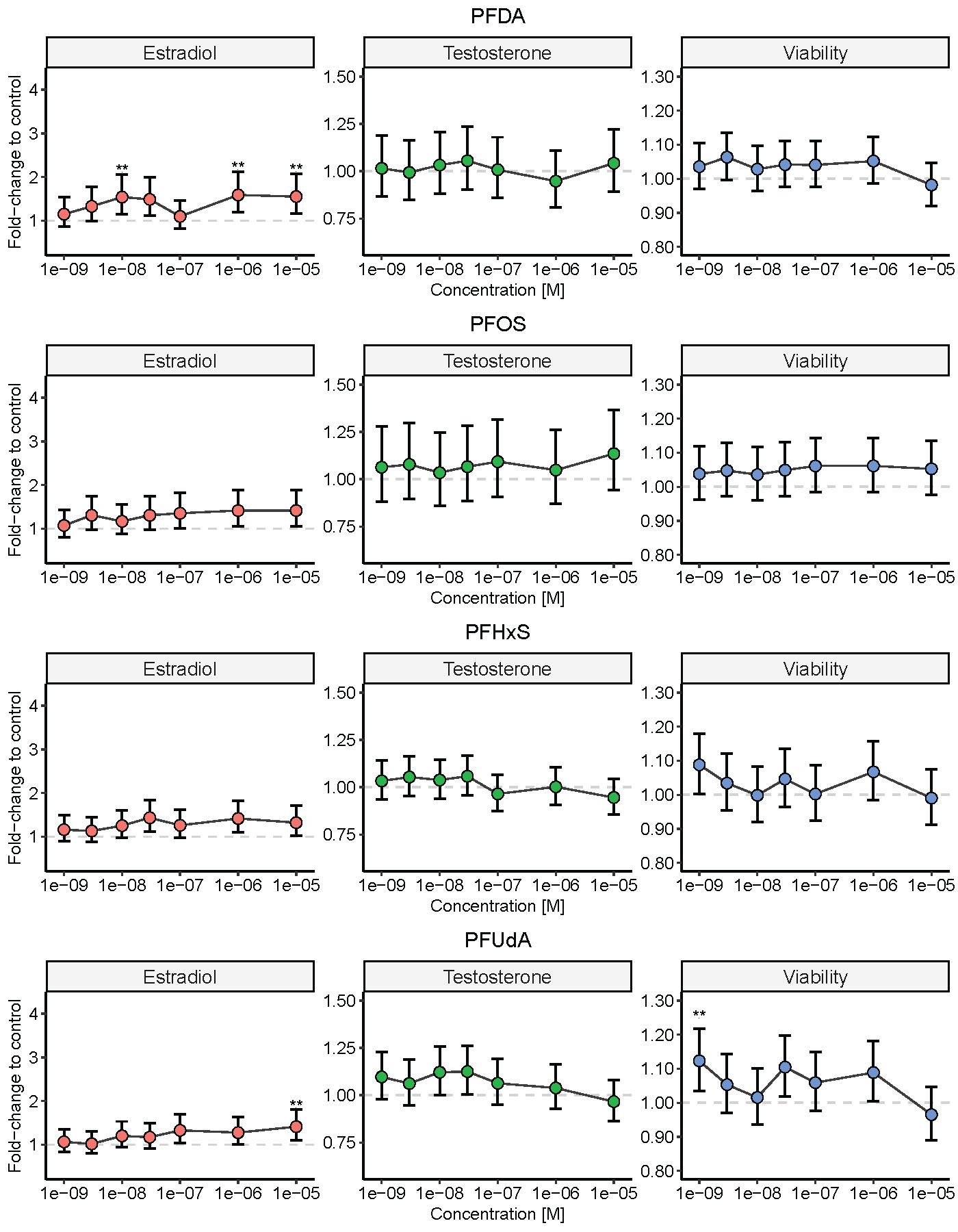


***Figure S10.*** *Screen of PFDA, PFOS, PFHxS, and PFUdA for effects on estradiol synthesis (red, left graph), testosterone synthesis (green, middle graph) and viability (blue, right graph) in H295R cells treated for 48h with 8 different concentrations ranging between 1nM-10 µM. Results are presented as fold-change of vehicle control (0.1% DMSO). Values repredent the mean of three experiments, each in technical triplicates and plotted with ± 95% confidence interval (CI). Statistical significance was calculated by LMM + Dunnett’s’ test and indicated with stars (*p<0.05; **p<0.01; ***p<0.001).*
